# The interplay of intrinsic excitability and network topology in spatiotemporal pattern generation in neural networks

**DOI:** 10.1186/1471-2202-15-S1-O16

**Published:** 2014-07-21

**Authors:** James P Roach, Leonard M Sander, Michal R Zochowski

**Affiliations:** 1 Neuroscience Graduate Program, University of Michigan, Ann Arbor, MI 48109, USA; 2 Department of Physics, University of Michigan, Ann Arbor, MI 48109, USA

## 

**Figure 1:**
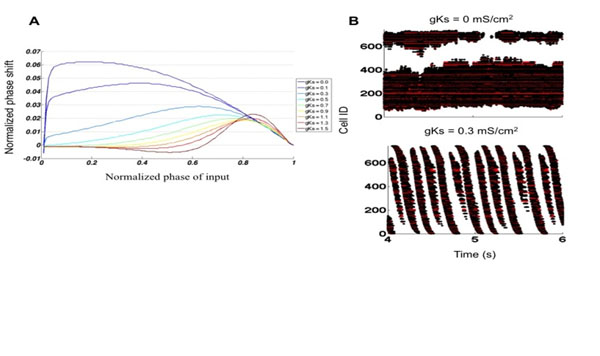
PRC induced changes in network dynamics. **A:** Increasing gKs shifts the PRC of the model neuron from type 1 at low values to type 2 at high values. **B:** Raster plots showing characteristic dynamics for networks at two different PRC types. Cells are sorted by distance from the origin in *xy* space and black dots represent excitatory action potentials and red dots indicate inhibitory action potentials

It is clear that spatiotemporal patterning in brain networks is a complex outcome of network physical connectivity and dynamical properties of interacting neurons, however characterization of this interaction remains elusive. These dynamical properties of the cells are affected/controlled by various neuromodulators secreted by the brain at various cognitive cycles or as a part of the response to the incoming stimuli. During sleep the brain cycles though distinct spatiotemporal patterns of neural activity. Acetylcholine (ACh) is a major regulatory factor of sleep states and plays an important role in the transition from slow wave sleep to waking or rapid eye movement sleep. Slow wave sleep is a slow oscillation in firing rate that travels through the cortical network and occurs when ACh levels are low [1]. At the cellular level, ACh causes changes in neural excitability by shifting the neural phase response curve (PRC) from type 2 to type 1 (Figure 1A)[2]. Previous modeling studies show that the shift of the PRC leads to a change from synchronous (type 2 PRC) to asynchronous (type 1 PRC), network dynamics while during low ACh levels networks display a high level of synchrony and network wide bursts [3]. As of yet the effects of intermediate cholinergic modulation have not been investigated. In this study, we use a Hodgkin-Huxley type model neuron which allows us to simulate different ACh levels and control a continuous transition from a type 1 to type 2 PRC [4]. We show that the PRC type of neurons drives different spatial patterns of activity within networks, with activity being highly localized for type 1 PRC neurons (Figure 1B) then quickly transitioning to wave dynamics as neurons are shifted to a type 2 PRC (Figure 1B). In networks composed of type 1 neurons, the region where activity is localized is defined by heterogeneities in network structure, with as little as a 1% increase in synaptic strength being sufficient to define the location of high activity. Additionally, the highly active zone is the origin of traveling waves in type 2 networks. When in the wave regime, decreasing cholinergic modulation of the PRC increases the speed that waves travel across the network. In summary, the precise character of frequency dynamics is governed by the interplay between network structure and the intrinsic excitability of component neurons. Expanding upon our results, we argue **(1)** that the intrinsic excitability of neurons shapes how activity spreads though a network and **(2)** that the focal point of traveling waves during slow wave sleep is a region selected for by synaptic potentiation.
